# Twisting the End Game: How Telomere Chromatin Modifications Shape Telomere Maintenance

**DOI:** 10.1111/acel.70694

**Published:** 2026-09-06

**Authors:** Jie Wang, Yuehui Su, Chao Chen, Yueying Li, Jiajia Han, Liping Du, Liyun Zheng, Ya‐Long Dang, Ying Peng

**Affiliations:** ^1^ Department of Ophthalmology The First Affiliated Hospital of Zhengzhou University Zhengzhou Henan China; ^2^ Sanmenxia Central Hospital Henan University of Science and Technology Sanmenxia Henan China; ^3^ Sanmenxia Eye Hospital Henan International Joint Laboratory of Glaucoma Aqueous Humor Drainage Sanmenxia Henan China; ^4^ Henan Institute of Medical and Pharmaceutical Sciences Zhengzhou University Zhengzhou Henan China; ^5^ Department of Gynecology The First Affiliated Hospital of Zhengzhou University Zhengzhou Henan China; ^6^ Department of Critical Care Medicine Henan Provincial People's Hospital, Zhengzhou University People's Hospital Zhengzhou Henan China; ^7^ Department of Infectious Diseases Henan Provincial People's Hospital, Zhengzhou University People's Hospital Zhengzhou Henan China; ^8^ Henan International Joint Research Laboratory for Ocular Immunology and Retinal Injury Repair, Department of Ophthalmology, Henan Province Eye Hospital The First Affiliated Hospital of Zhengzhou University Zhengzhou Henan China

## Abstract

Cell division inevitably shortens telomeric DNA owing to the end‐replication problem. Eukaryotic chromosomes possess specialized telomere structures to maintain genomic stability. In most proliferative cells, telomerase adds telomeric repeats during S‐phase. In differentiated cells where telomerase is silenced, telomeres shorten progressively, thereby compromising genomic integrity. Consequently, cancer cells universally activate alternative telomere maintenance mechanisms during malignant transformation: ~80% reactivate telomerase, while a portion of the rest rely on BIR (break‐induced replication)–mediated homologous recombination‐based ALT (alternative lengthening of telomeres). Although these mechanisms are stable once established, the initial determinants influencing a cancer cell's choice remain poorly understood. This review discusses recent molecular insights into how telomeric chromatin properties profoundly impact this choice. After briefly introducing telomere chromatin characteristics and key players in its maintenance and dynamics, we discuss the mechanisms by which cancer cells acquire distinct telomere replication capabilities. In particular, we present an in‐depth analysis linking telomere heterochromatin status to ALT. Furthermore, based on recent advances, we propose a coupled feedforward loop model explaining how the ALT state becomes “locked in” once initiated. Finally, we offer novel perspectives on rational, telomere‐centric therapeutic interventions for ALT‐positive cancers, focusing on strategies designed to disrupt such feedforward loops by manipulating telomeric chromatin structure.

## Graphical abstract

Chromatin modifications on telomeres are linked to the mode of telomere DNA replication. A coupled feedforward loop model is proposed based on these to lock the ALT cancer cells in this alternative mode of telomere replication.

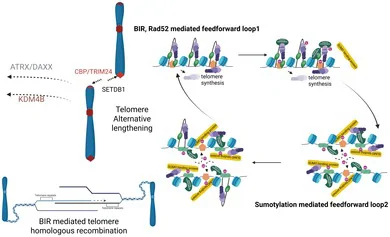

## 1 Discovery of Telomere Heterochromatin Through Position Effect Variegation Phenomenon

Long before the nucleosome was identified as the fundamental unit of chromatin, early cytologists like Emil Heitz observed, nearly a century ago, that certain regions of mitotic chromosomes stained more densely than others. Such cytological regions were described as “heterochromatin,” which were proposed to be enriched in parts of the genome without genes or associated with functionally “passive” regions (Chen et al. 2024). Such proposition led to the recognition that telomeres are composed of condensed constitutive heterochromatin, a state crucial not only for end‐protection but also for silencing genes in adjacent subtelomeric regions which would express normally in their native chromosomal location (Schoeftner and Blasco 2009). The latter telomeric heterochromatin‐associated gene silencing was first unveiled by early genetics pioneers through the discovery of a phenomenon known as PEV (position effect variegation). This occurs when a euchromatic gene—such as the white‐eye gene in fruit flies—is translocated near a chromosomal end (telomere) (Muller 1930). In flies with such translocations, the white gene locus is stochastically silenced via heterochromatin spreading in photoreceptor cells, resulting in the characteristic variegated eye‐color pattern that gave the phenomenon its name.

**FIGURE 1:**
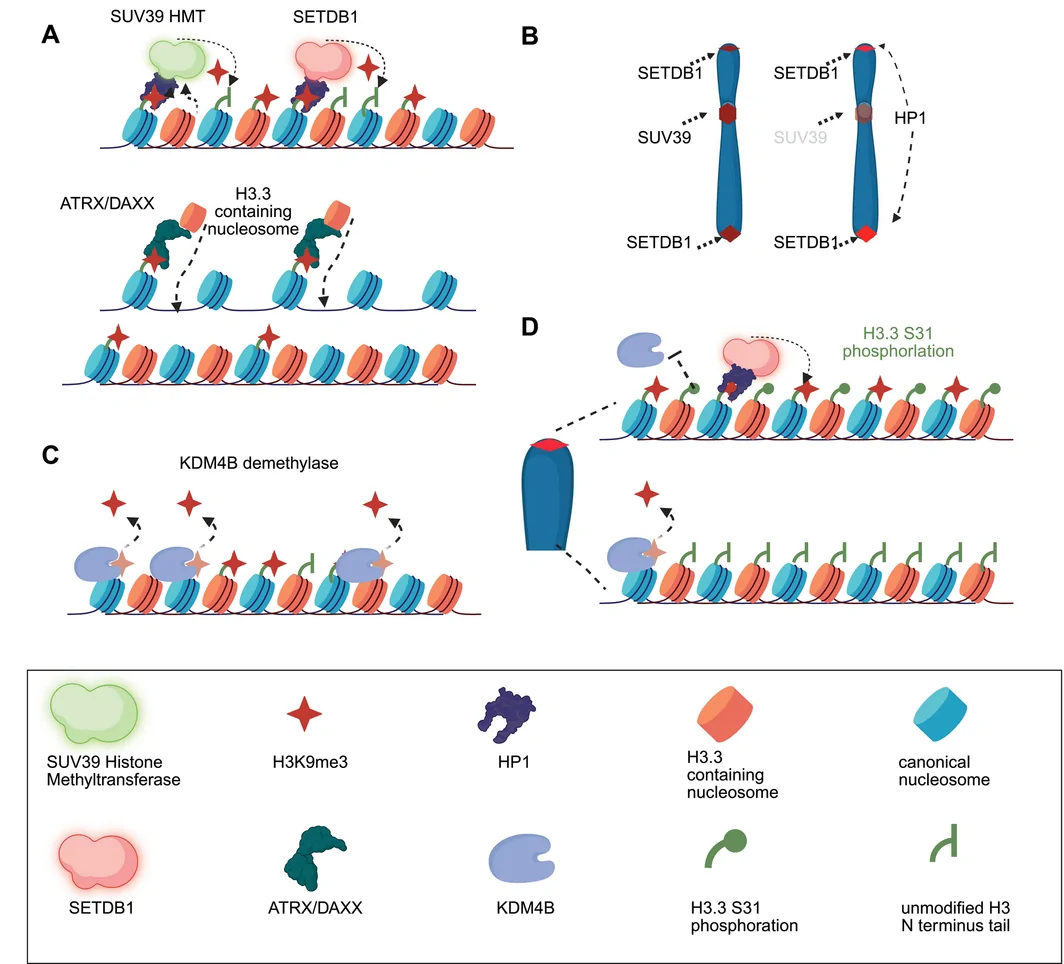
Human telomere heterochromatin is dynamically regulated by the coordinated actions of histone modification “writers,” “readers,” and “erasers.” (A) Writer: In human cells, the core writer enzymes for H3K9me3 modification include SUV39 family histone methyltransferases (HMTs) and SETDB1. SUV39 family HMTs (SUV39H1 and SUV39H2) are predominantly responsible for H3K9me3 modification at pericentromeres, while SETDB1 serves as the major H3K9me3 writer at telomeres. The specificity of SUV39H for pericentromeres arises from its chromodomain acting as a reader of dual H3K9me3 and H3K14ub modifications. Reader: The core H3K9me3 readers include HP1 and the ATRX/DAXX chaperone complex. HP1 binds H3K9me3 via its N‐terminal chromodomain, and recruits additional SUV39 family HMTs. ATRX recognizes H3K9me3 through its cysteine‐rich ADD domain. Together with DAXX, it facilitates the replication‐independent deposition of H3.3‐containing nucleosomes at telomeres. (B) A “remote tag‐of‐war” exists between pericentromeric and telomeric heterochromatins. Human heterochromatin at pericentromeric region and telomeres are mainly deposited by distinct HMT writers (SUV39H and SETDB1 respectively). In cells with SUV39H knockout (KO), the loss of pericentromeric heterochromatin is associated with a concurrent gain of telomeric heterochromatin, potentially through the transfer of the H3K9me3 reader HP1 from pericentromeres to telomeres. (C) Eraser: KDM4B functions as the key eraser of heterochromatin histone mark with its H3K9 demethylase activity. (D) KDM4B demethylase activity is tightly regulated by H3.3 S31 phosphorylation. Therefore, around telomeres its eraser activity is blocked by the presence of phosphorylated H3.3 at serine 31 (H3.3 S31P) (top), whereas at euchromatin, the absence of H3.3 S31P enables KDM4B to be fully functional, removing sporadic H3K9me3 modifications on the chromosome proper (bottom).

PEV has served as a powerful genetic tool for identifying key regulators of heterochromatin (Elgin and Reuter 2013), primarily through screens for mutations that modify PEV phenotypes. These collective efforts led to the identification of many key molecular players that shape heterochromatin. For example, the first heterochromatin‐specific protein, HP1, was discovered in *Drosophila* as a strong suppressor of PEV (Eissenberg et al. 1990). Another PEV suppressor identified from such screens is Su(var)3–9 (suppressor of variegation, on chromosome 3, #9). This gene encodes a critical histone methyltransferase that specifically methylates histone H3 at lysine 9 (H3K9) (Aagaard et al. 1999; Tschiersch et al. 1994; Schotta et al. 2002). In Su(var)3–9 null mutants, H3K9me3 at heterochromatin is drastically reduced, demonstrating that Su(var)3–9 is the major heterochromatin‐specific H3K9 methyltransferase. The function of this enzyme is highly conserved, as evidenced by subsequent studies in yeast and mice. Consequently, nucleosomes marked by high levels of H3K9me3 are now a definitive hallmark of heterochromatin. Genomic regions such as telomeres and pericentromeres represent classic examples of constitutive heterochromatin. Once established, a self‐reinforcing positive feedback loop maintains the H3K9me3 modification, locking these domains into a stably silenced state (Grewal 2023). The positive feedback loop is primarily mediated by HP1, the first‐identified reader specific for heterochromatin. HP1's N‐terminal chromodomain binds tightly to the H3K9me3 mark on histone H3 tails. Once recruited to H3K9me3‐enriched heterochromatin, HP1 forms dimers that bridge adjacent nucleosomes (Chow et al. 2018; Canzio et al. 2013; Machida et al. 2018; Sokolova et al. 2024). Simultaneously, its C‐terminal chromo shadow domain efficiently recruits more Su(var)3–9 methyltransferase (Thiru et al. 2004) (Figure 1A). This recruitment ensures that newly incorporated histones are continuously methylated at H3K9, thereby maintaining the high H3K9me3 levels characteristic of heterochromatin (Cheutin et al. 2003; Festenstein et al. 2003). The integrity of this feedback mechanism is central to heterochromatin maintenance and spreading, particularly into subtelomeric regions. This is evidenced by the extreme sensitivity of PEV to genetic perturbations in either HP1 or Su(var)3–9 (Schotta et al. 2002; Wustmann et al. 1989; Locke et al. 1988). Furthermore, studies in model organisms like yeast and flies have revealed that RNA interference (RNAi) pathways and specific DNA sequences are also crucial for sustaining heterochromatin (Cheng and Gartenberg 2000; Brower‐Toland et al. 2007; Volpe et al. 2002; Wei et al. 2021). Notably, genetic screens for PEV modifiers have also unveiled active processes that constrain heterochromatin spread. For instance, histone acetylation and phosphorylation counterbalance the spillover of H3K9me3 marks from telomeres into neighboring euchromatin. This establishes a dynamic equilibrium between constitutive telomeric heterochromatin and the adjacent chromosomal arm in euchromatin (Wakimoto 1998; Ebert et al. 2004). Similar to observations in flies and yeast, TPE (telomere position effect) has also been observed in human cells. This suggests that a repressive, self‐propagating chromatin state at telomeres is a conserved feature from unicellular eukaryotes like yeast to humans (Tennen et al. 2011).

## 2 The Characteristics of Human Telomere Heterochromatin

### 2.1 Shelterin Complex, Human Telomere Nucleosome Organization, Histone Variant H3.3 Deposition and TERRA Non Coding RNA

Mammalian telomeres comprise tracts of predominantly TTAGGG DNA repeats bound by a protective protein complex named Shelterin, which consists of six subunits: TRF1, TRF2, POT1, RAP1, TIN2, and TPP1. The loss of any Shelterin component leads to telomere uncapping and activation of DNA damage response pathways (de Lange 2005). As core components, TRF1 and TRF2 directly bind double‐stranded hexameric DNA repeats (5′‐TTAGGG‐3′). Critically, when telomeres shorten below a critical length, Shelterin fails to maintain its protective association, triggering irreversible DNA damage signaling and cell cycle arrest (Hemann et al. 2001; van Steensel and de Lange 1997). Shelterin actively suppresses two major DNA damage response pathways to protect functional telomeres. The POT1 subunit binds the single‐stranded telomeric overhang and prevents its recognition by the ATR‐ATRIP sensor, thereby blocking the activation of the ATR kinase cascade that would otherwise induce cell cycle arrest (Hockemeyer et al. 2005; Denchi and de Lange 2007). Similarly, TRF2 inhibits the activation of the ATM kinase pathway triggered by double‐strand breaks at chromosome ends (Denchi and de Lange 2007).

Despite the sequence‐specific binding of Shelterin, mammalian telomeric DNA is predominantly packaged into nucleosomes. How nucleosomes and Shelterin cooperate to form a coherent capping structure remains an open question. Notably, telomeric chromatin exhibits a much shorter nucleosome repeat length than typical euchromatin (Makarov et al. 1993; Tommerup et al. 1994; Fajkus and Trifonov 2001; Episkopou et al. 2014), which led to the proposal of a tightly packed columnar nucleosome model (Fajkus and Trifonov 2001). Cryo‐EM (Soman et al. 2022) studies of reconstituted telomeric chromatin have visualized this compact state, showing DNA continuously wound around stacked histone cores without the typical nucleosomal gaps. Intriguingly, an alternative “open” nucleosome arrangement coexists within the same fibers (Soman et al. 2022), where adjacent nucleosomes are tilted, exposing short DNA gaps. This structural flexibility may allow Shelterin to bind its recognition sequences without necessarily evicting nucleosomes. Furthermore, Shelterin promotes higher‐order chromatin compaction by facilitating intramolecular interactions between adjacent telomeric fibers (Soman et al. 2022).

Although telomere DNA replication by telomerase is confined to S phase, telomeric nucleosomes undergo considerable turnover during interphase. This replication‐independent turnover is primarily mediated by the histone variant H3.3. Two major cellular chaperone complexes deposit H3.3 during the physiological state: the HIRA complex targets active genes, while the ATRX/DAXX complex specifically enriches H3.3 at heterochromatic regions like telomeres and pericentromeres (Lewis et al. 2010). Compared to canonical H3 nucleosomes, H3.3‐containing nucleosomes are intrinsically less stable and more prone to losing H2A/H2B dimers (Jin and Felsenfeld 2007). This inherent lability may make H3.3 nucleosomes particularly suited to intermingle with the Shelterin complex or to maintain a dynamic and accessible chromatin architecture at telomeres. In addition, H3.3‐containing nucleosomes are known to be the major substrate for H3K9me3 modification at telomeres, and are essential for maintaining the telomeric heterochromatin hallmark (Udugama et al. 2015).

Telomeres are also sites of active transcription (Yu et al. 2014; Nergadze et al. 2009). Most human chromosome ends are associated with CpG‐rich subtelomeric promoters located directly upstream of the TTAGGG repeats (Nergadze et al. 2009). These promoters drive the expression of long noncoding TERRA (telomeric repeat‐containing RNA) (Azzalin et al. 2007). TERRA plays a multifaceted role in heterochromatin regulation: it promotes the establishment of H3K9me3 marks and the recruitment of HP1 to telomeres (Deng et al. 2009), and it may also influence DNA methylation at subtelomeric regions. Paradoxically, TERRA transcription is associated with the recruitment of the MLL1 histone methyltransferase (Caslini et al. 2009), which catalyzes the deposition of the activating H3K4me3 mark, thereby counteracting repressive H3K9me3. Thus, TERRA is involved in a sophisticated negative feedback loop that modulates both its own transcription and the dynamic equilibrium of telomeric heterochromatin. In addition, TERRA promotes R‐loop (RNA–DNA hybrid loop) formation on telomeres by binding to telomeric DNA, which is a key intermediate promoting the ALT mode of telomere maintenance (Bhargava et al. 2022; Namous and Vemuganti 2025). RAD51 recombinase facilitates R‐loop formation and its subsequent resolution by physically binding to TERRAs (Feretzaki et al. 2020; Kaminski et al. 2022). Therefore, TERRA expression was both necessary and directly required to activate break‐induced replication in ALT cells (Silva et al. 2021).

### 2.2 Players in Human Telomere Biology: Writers, Readers, and Erasers

The establishment, maintenance, and removal of histone modifications are governed by three key classes of mediators: writers, readers, and erasers. Among the writers, several mammalian histone methyltransferases target H3K9, including SUV39H1, SUV39H2, SETDB1, SETDB2, G9a, and GLP. Notably, G9a and GLP primarily catalyze mono‐ and di‐methylation of H3K9 and are thus not directly involved in constitutive heterochromatin formation (Shinkai and Tachibana 2011). In contrast, SUV39 and SETDB family proteins are proficient H3K9me3 methyltransferases. In invertebrates, SUV39 serves as the major H3K9me3 writer for both centromeric and telomeric heterochromatin. In human cells, however, this role appears largely compartmentalized. SUV39H1 and SUV39H2 are both responsible for H3K9me3 deposition at pericentromeric heterochromatin (Peters et al. 2001; Benetti et al. 2007; García‐Cao et al. 2004), while SETDB1 predominantly deposits the H3K9me3 mark at telomeres (Gauchier et al. 2019). Such a clear division of labor between distinct HMTs (histone methyltransferases) might be blurred under specific pathological situations, depending on the ad hoc recruitment mechanisms when the telomere homeostasis is tilted away from equilibrium (Schoeftner and Blasco 2009; Porro et al. 2014). Although chromatin specific proteomics confirms the differential association of SUV39H and SETDB1 with pericentromeres and telomeres (Gauchier et al. 2019), the precise basis for this functional diversification remains largely unresolved. Recent studies on pericentromeric heterochromatin reveal that SUV39H acts as a reader for dual H3K9me3 and H3K14ub modifications, with its specific targeting potentially mediated through binding to H3K14ub, a mark catalyzed by the pericentromere‐localized E3 ligase G2E3 (Huang et al. 2025). Whether a similar combinatorial set of histone codes directs SETDB1 to telomeres remains to be explored. Intriguingly, a functional antagonism exists between pericentromeric and telomeric heterochromatin (Figure 1B). For instance, in SUV39H‐double knockout cells, the loss of pericentromeric heterochromatin is associated with a concurrent gain of telomeric heterochromatin, potentially due to the redistribution of the reader protein HP1 (Gauchier et al. 2019). Conversely, loss of SETDB1 reduces telomeric heterochromatin but does not increase TERRA transcription as predicted; instead, TERRA appears as shortened RNAs, suggesting premature transcription termination (Gauchier et al. 2019).

**FIGURE 2:**
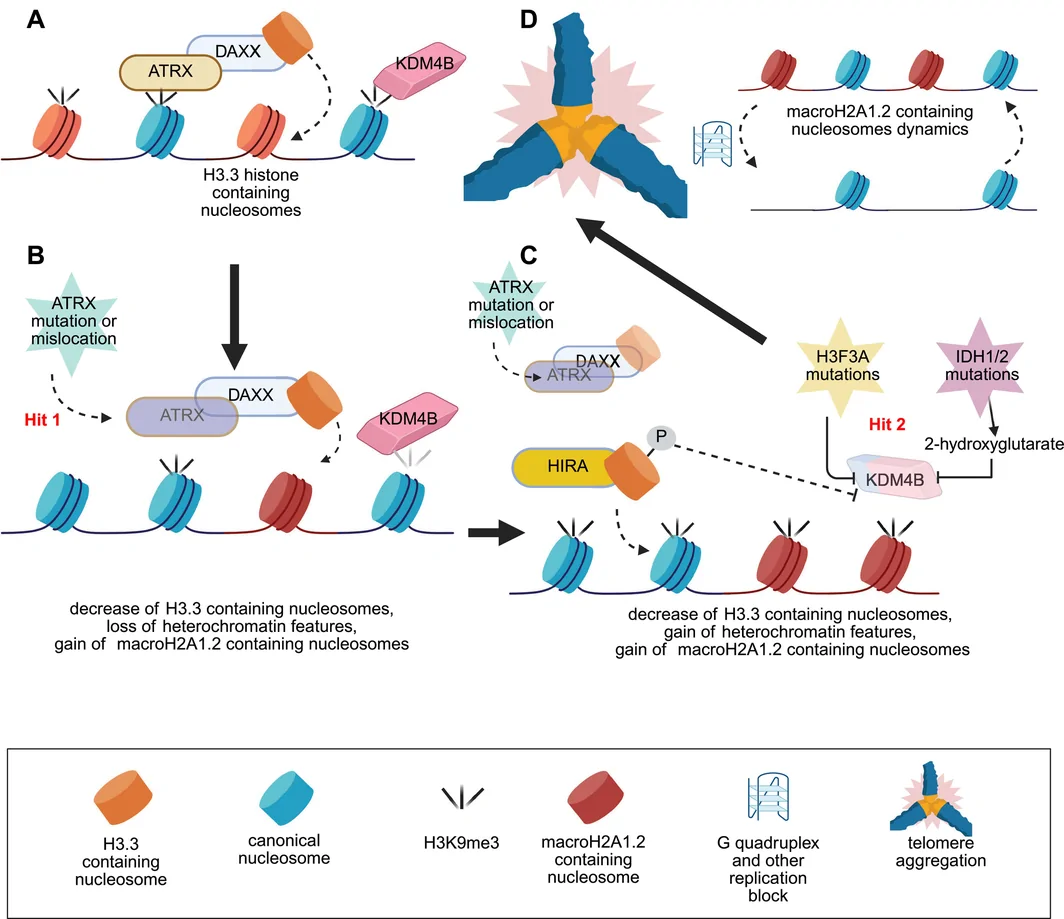
The “Two‐Hits” model of ALT pathway activation via telomere chromatin remodeling. (A) Schematic representation of the steady‐state telomeric chromatin in non‐ALT cells. The ATRX/DAXX chaperone complex deposits histone variant H3.3 containing nucleosomes (orange) at telomeres. The H3K9me3 demethylase KDM4B maintains heterochromatin homeostasis by removing excessive H3K9me3 marks. (B) The first hit in ALT cells: Loss or mislocalization of ATRX/DAXX (dark gray ATRX box). Without the default chaperone, this leads to reduced deposition of H3.3 containing nucleosomes (orange) and accumulation of nucleosomes containing the histone variant macroH2A1.2 (magenta). (C) The second hit in ALT cells: In the presence of DNA damage, histone chaperone HIRA can act on telomeres depositing H3.3 containing nucleosomes enriched with H3.3S31ph modification. H3.3S31ph strongly inhibits local KDM4B demethylase activity, which can be further inactivated with one of the possible second genetic hits: Either H3F3A mutations (e.g., G34R/V) or IDH1/2 mutations that produce the oncometabolite 2‐hydroxyglutarate as a competitive KDM inhibitor. Loss of KDM4B activity leads to accumulation of higher steady‐state level of H3K9me3 at telomeres. (D) When experiencing telomeric replication stress (e.g., G‐quadruplex or other replication block), cells harboring “two‐hits” alterations induce dynamic turnover of macroH2A1.2‐containing nucleosomes during the stress induction/relief cycles. The resultant chromatin remodeling favors processes initiating ALT mode of telomere maintenance.

There are multiple heterochromatin‐associated proteins at telomeres, many of which are so called histone reader proteins binding directly to H3K9me3 (Figure 1). The most well‐established H3K9me3 reader is HP1, which specifically recognizes trimethylated H3K9 through its chromodomain. As discussed previously, HP1 is a critical component in the positive feedback loop maintaining H3K9me3 marks on heterochromatin. In addition, ATRX (alpha‐thalassemia/mental retardation, X‐linked), as the core component of the ATRX/DAXX chaperone complex responsible for H3.3 deposition at heterochromatin, also functions as a critical H3K9me3 reader (Figure 1). Its cysteine‐rich ADD (ATRX‐DNMT3‐DNMT3L) domain forms an atypical composite H3K9me3‐binding pocket, where alpha‐thalassemia/mental retardation, X‐linked syndrome associated pathogenic mutations are enriched (Iwase et al. 2011). Conversely, in SETDB1‐knockout cells, ATRX was excluded from telomeres and mostly colocalized with pericentromeres (Gauchier et al. 2019). Therefore, H3.3 deposition at telomeres is in turn dependent on their heterochromatin status, mediated by the reader function of ATRX (Figure 2). On the other hand, H3.3 is a major substrate for H3K9me3 modifications, and therefore indispensable for maintaining the integrity of mammalian telomeric heterochromatin (Udugama et al. 2015).

In contrast to H3K9me3 writers (methyltransferases), KDMs (histone lysine demethylases) are responsible for removing heterochromatin marks (Figure 1C) (Gray et al. 2025). Members of the KDM4 family possess two tandem Jumonji demethylase domains (JmjN and JmjC), which are capable of demethylating trimethylated lysine residues. H3K9me3‐enriched heterochromatic regions utilize KDM4A to remove H3K9me3 marks during S phase, enabling adequate chromatin accessibility for subsequent DNA replication (Black et al. 2010). Among KDM4 family members, however, KDM4B demethylates H3K9me3 most effectively, with additional reactivity for H3K36me3 demethylation (Whetstine et al. 2006; Klose et al. 2006; Cloos et al. 2006; Chen et al. 2006; Udugama et al. 2021), and is believed to be the major KDM maintaining telomere heterochromatin homeostasis (Udugama et al. 2021). Most KDM4 proteins contain DTDs and two PHD domains, both of which are dispensable for catalytic activity but critical for substrate recognition. DTDs from KDM4A or KDM4B bind strongly to H3K23me3 (Su et al. 2016), a histone mark with limited functional understanding. In addition, KDM4B binds strongly to the unmodified H3.3 N‐tail deprived of S31 phosphorylation. The serine 31 residue in H3.3 (its only divergent residue from canonical H3 beyond its globular core domain) is highly conserved from yeast to mammals and can be phosphorylated. Upon S31 phosphorylation, the H3.3 N‐terminal tail strongly inhibits KDM4B lysine demethylase activity. Therefore, a strong enrichment of H3.3 S31P (phosphorylation of serine 31 on histone variant H3.3) modification on heterochromatins, including telomeres, is thought to restrict KDM4B‐mediated H3K9 demethylation at telomeres (Udugama et al. 2022) (Figure 1D), maintaining a high local density of H3K9me3 marks deposited by the HP1/SETDB1 positive feedback at steady state. In mouse ES cells with a knock‐in phosphor‐mimic H3.3 S31E mutation, both telomere heterochromatin formation and ATRX binding are significantly boosted. Simultaneously, the consequential complete suppression of KDM4B binding and the loss of demethylation activity lead to increased nucleosome occupancy and inaccessibility in telomeres (Udugama et al. 2022) (Figure 1D).

Besides histone methylation, chromatin poly(ADP‐ribosyl)ation is also reported to be involved in telomere physiology. DNA‐dependent ADP‐ribosyl transferases (including PARP1 and PARP2) are activated by DNA breaks. PARP enzymatic activity is particularly critical for the telomeric function of the chromatin assembly factor HIRA, which is normally not responsible for nucleosome deposition at telomeres. However, when the default telomere associated H3.3 chaperone ATRX/DAXX is defective and replication stress accumulates subsequently, HIRA acts as a poly(ADP‐ribose) reader, thereby allowing the telomere deposition of histone H3.3 as a salvage pathway (Bhargava et al. 2022; Muoio and Fouquerel 2025; Sung 2025; Lynskey et al. 2024). It is noteworthy that poly(ADP‐ribose) marks on telomeres are not only a passive response toward local DNA breaks, but are also functionally important under given physiological circumstances. Inhibition of PARG, which erases the poly(ADP‐ribose) marks on chromatin, leads to reductions of ALT‐related features and shortens telomeres in ALT cancer cells (Bhargava et al. 2022). Therefore, cancer cells relying on the ALT mode of telomeric replication are in general more sensitive to PARPi (PARP inhibitor) within the context of a sublethal dosage of replication stress (Laemmerer et al. 2025).

### 2.3 Telomerase vs. ALT Replication Mode for Telomere Replication

Telomere replication takes place within the framework of telomere chromatin. Studies in humans have revealed a combination of heterochromatic and euchromatic markers, along with histone modifications linked to active transcription across various cell models. This suggests that the chromatin state of telomeres may affect how new telomeric DNA sequences are added to existing telomeres.

In all untransformed stem cells and most human cancer cells, telomerase is activated to facilitate telomere replication (Barthel et al. 2017). Since telomerase needs sufficient access to telomeric DNA, chromatin accessibility is essential for telomere synthesis. In human cancer cell lines that depend on telomerase, additional heterochromatin characteristics can be acquired on telomeres through TRF1‐HP1 tethering, likely involving an increase in H3K9me3 modifications, which further stabilize telomere structures. This accumulation of heterochromatin features leads to a gradual decline in telomere replication over successive cell cycles, even under conditions of telomere replication stress (Chow et al. 2018), indicating that telomeres with excessive heterochromatin are poor substrates for telomerase‐based replication.

Approximately 20% of human cancers do not reactivate telomerase expression. Instead, many of these cells use the ALT mechanism to overcome telomere shortening and support continuous cell division (Barthel et al. 2017). Break‐induced telomere synthesis begins with the resection of a damaged telomere end, where the guanosine‐rich strand is nicked to create a single‐stranded DNA segment that can pair with a homologous sequence from another telomere, forming a D‐loop (displacement loop). These strand invasion intermediates become substrates for telomeric replication via the BTR dissolvase complex (BLM‐TOP3A‐RMI), which initiates rapid and extensive DNA polymerase δ‐dependent telomere synthesis followed by dissolution, without an overall exchange of telomeric DNA (Dilley et al. 2016; Sobinoff et al. 2017). Alternatively, the same recombination intermediate can be processed by the SLX4‐SLX1‐ERCC4 complex, achieving replication stress resolution without extending the telomere (Sobinoff et al. 2017), thus supporting ALT‐based telomere synthesis. Therefore, ALT telomeres maintain a delicate balance between “dissolution”—mediated by the BTR complex and resulting in telomere extension without exchange—and “resolution”—mediated by the SLX4 complex and resulting in telomere exchange events with abortive extension. Both pathways address telomeric replication stress (Sobinoff et al. 2017). Alternatively, stalled replication fork triggering replication stress can also be eased temporarily without strand invasion. ATP‐dependent DNA translocases FANCM and SMARCAL1 can block the BTR‐dependent formation of strand invasion intermediates but instead push replication fork regression to fix the DNA damage. In ALT cells, both DNA translocases FANCM and SMARCAL1 are maintained at a high level so as to ease the excessive replication stress during BTR‐mediated telomere homologous recombination (Lu et al. 2019; Halder et al. 2022; Fielden et al. 2025; Bansbach et al. 2009).

Physically, ALT occurs exclusively within APBs (ALT‐associated promyelocytic leukemia bodies), where multiple independent telomeres cluster into an intertwined focus. Unlike telomerase‐mediated replication during the S phase, most APB‐associated telomere synthesis takes place in the G2 phase of the cell cycle (Dilley et al. 2016). APBs not only bring homologous telomere sequences into close proximity but also concentrate the necessary recombination enzymes on the clustered telomeres, facilitating efficient telomere synthesis.

While pioneering work by the Jan Karlseder group a decade ago demonstrated that ALT can be a direct consequence of histone dysfunction through depletion of histone chaperones ASF1a and ASF1b (O'Sullivan et al. 2014), it remains under intense debate how telomere chromatin status might influence the choice of telomere replication in ALT mode. For instance, ALT cells lacking SETDB1 (a key H3K9me3 methyltransferase for telomeric heterochromatin formation) showed decreased binding of several recombination factors related to ALT, whereas these factors were more enriched at telomeres in Suv39h (a key H3K9me3 methyltransferase for pericentromeric heterochromatin formation) knockout cells. Therefore, telomere heterochromatin seems to promote the appearance of ALT features. However, the recruitment of ATRX/DAXX complexes to telomeres relies on telomeric loading of the H3.3 histone variant and subsequent deposition of H3K9me3 marks; therefore, at least some heterochromatin features are needed to counteract the ALT process mediated by ATRX/DAXX. Accordingly, ATRX cannot prevent telomere recombination when telomeres lose their heterochromatic features (Gauchier et al. 2019). On the contrary, when the H3K9me3 eraser KDM4B is inhibited by introducing a phospho‐mimic mutation at H3.3 S31, telomeres become excessively heterochromatic, yet ALT does not occur spontaneously. Instead, telomeres subsequently shorten along with increased telomeric DNA damage and a lack of efficient repair mechanisms (Udugama et al. 2022). Therefore, both excessive heterochromatin and the absence of heterochromatin can disrupt ALT activity and lead to compromised telomere integrity.

## 3 Linking Telomere Heterochromatin Status and Alternative Telomere Lengthening

### 3.1 ALT Requires a “Two‐Hits” Damage on the Telomere Chromatin

The ALT pathway is adopted in a significant portion of human cancers, with high prevalence in gliomas, pancreatic neuroendocrine tumors, osteosarcomas, and soft‐tissue sarcomas (Heaphy, de Wilde, et al. 2011; Heaphy, Subhawong, et al. 2011). Analysis of mutational profiles in ALT‐positive human cancer biopsies reveals a distinct mutation pattern. Notably, inactivating mutations in the ATRX and DAXX genes are strongly associated with ALT‐positive cancers, including pediatric glioblastoma, pancreatic neuroendocrine tumors, and sarcomas (Schwartzentruber et al. 2012; Jiao et al. 2011; Lovejoy et al. 2012; Liau et al. 2015). In rare cases of biopsy samples with ALT telomere features but without detectable ATRX/DAXX mutations, the cancer cells often lost the nuclear localization of either ATRX or DAXX (Heaphy, de Wilde, et al. 2011) (Figure 2B), indicating an almost universal link between defects in ATRX/DAXX chaperone function and ALT. Transient ATRX expression in ATRX‐null ALT cells represses ALT activity (Clynes et al. 2015; Napier et al. 2015), firmly establishing a causal relationship between ATRX/DAXX loss and ALT telomere replication mode. Since ATRX/DAXX complex acts as the default H3.3‐specific histone chaperone responsible for a majority of replication‐independent chromatin assembly at telomeres (Lewis et al. 2010), ATRX‐DAXX deficiency directly disrupts DAXX‐mediated deposition of H3.3 at ALT telomeres. In their place, specialized histone chaperone HIRA can be mobilized with the help of chromatin poly(ADP‐ribosyl)ation induced by local DNA replication stress to execute H3.3 containing nucleosome loading, facilitating continuous telomeric nucleosome turnover in ALT cells (Lynskey et al. 2024; Hoang et al. 2020). Therefore, depletion of HIRA sensitizes death of ATRX‐DAXX‐mutated ALT cancer cells, which can be rescued by restoring ATRX function (Hoang et al. 2020). Subsequently, it has been elucidated that ATRX/DAXX loss promotes persistent telomere cohesion and recombination between sister telomeres before mitosis, while suppressing inappropriate recombinations between non‐sister telomeres (Ramamoorthy and Smith 2015). Recently it has been found that in high‐grade pediatric osteosarcomas, a majority of ALT‐positive cancer cases are driven by genomic amplification containing TOP3A locus rather than by ATRX mutation (de Nonneville et al. 2022). Therefore, TOP3A overexpression might override the requirement for ATRX in restraining ALT. Although loss of ATRX/DAXX is nearly always necessary for the ALT replication mode, knockdown or knockout of ATRX/DAXX alone does not induce the ALT phenotype. This has led to the hypothesis that a “two‐hits” process is required for cells to adopt ALT. Indeed, a majority of ATRX‐mutated GBMs (glioblastoma multiforme) have concomitant additional mutations in the checkpoint regulator TP53, and either H3F3A (encoding histone H3.3) or IDH1 (isocitrate dehydrogenase 1) (Figure 2C). Notably, mutations in H3.3 and IDH1 are almost mutually exclusive, implying that they independently converge on a common pathway (Udugama et al. 2021). The most frequent H3F3A mutations (K27M, G34R, and G34V) inhibit KDM4 demethylases (Voon et al. 2018), while most IDH1 mutations (such as R132H) generate the metabolite 2‐hydroxyglutarate, which inhibits alpha‐ketoglutarate‐dependent dioxygenases, including the KDM4 enzymes (Xu et al. 2011; Couteau et al. 2025) (Figure 2C). This suggests that a common histone eraser might be the key target for additional ALT‐associated mutations. Among potential candidates, KDM4B is the most prominent demethylase at human telomeres, as ALT can be effectively induced in cell models by simultaneously depleting ATRX and KDM4B (Udugama et al. 2021) (Figure 2).

Such a two‐hits hypothesis pinpointed that ALT telomeres require specific chromatin features, involving a combination of the absence of the ATRX/DAXX chaperone complex and the presence of stable H3K9me3 heterochromatin. Since the ATRX/DAXX complex is primarily known for depositing H3.3‐containing nucleosomes, the lack of either ATRX or H3.3 alone results in lower telomeric H3K9me3 levels and makes DNA more sensitive to replication stress (Udugama et al. 2015). This suggests H3.3 to be a major substrate for SETDB1‐mediated H3K9 hyper‐methylation. However, in ATRX‐null cells with normal KDM4B function, telomeric H3K9me3 levels may remain too low to support the ALT process. A second mutation causing loss of KDM4B activity could raise telomeric H3K9me3 to an appropriate steady‐state level. Notably, in cells with functional ATRX and the H3.3 S31E phosphomimetic mutation, KDM4B activity is strongly inhibited in the context of H3.3‐containing, H3K9me3‐rich telomeric chromatin. In this extreme scenario, ALT also cannot proceed with excessive heterochromatin. Thus, the two‐hits process of ALT likely involves additional chromatin features of telomeres beyond the heterochromatin level. For example, the absence of ATRX/DAXX is also associated with the accumulation of nucleosomes containing the histone variant macroH2A1.2 (a histone H2A variant with almost three times the size of canonical counterpart, due to the presence of an evolutionarily conserved nonhistone macro domain) at telomeres, which helps protect fragile DNA against replication stress‐induced damage (Kim et al. 2018, 2019). Human ALT telomeres are highly enriched for macroH2A1.2 (Kim et al. 2019). However, during acute replication stress, a rapid and dynamic depletion of macroH2A1.2‐containing nucleosomes occurs specifically in ALT cells, indicating an crosstalk between telomeric macroH2A1.2‐containing nucleosomes and heterochromatin (Kim et al. 2019) (Figure 2D). Such stress‐induced, transient depletion of macroH2A1.2 on ALT telomeres, along with increased levels of trapped proteins on telomeres in ATRX‐deficient cells (Rose et al. 2023), may collectively contribute to the ALT‐permissive chromatin state. All of these events on permissive telomeric chromatin lead to enhanced MUS81 endonuclease (a critical structure‐specific cellular endonuclease that cleaves on 3′‐flaps and nicked Holliday junctions) activity, which is indispensable for the subsequent ALT process (Zeng et al. 2009; Pepe and West 2014; Gwon et al. 2014; Hua et al. 2022).

Despite the logically clear‐cut framework of such a “two‐hits” hypothesis to explain the major genetic requirements for a full spectrum of ALT mode of telomeric replication, tumor cases are highly heterogeneous with distinctive ALT‐like features. Therefore, great caution should be taken by clinicians when fitting every ATRX/DAXX mutated cancer case to such a rigorous framework. For example, ATRX‐mutation can be associated with other genetic lesions such as MN1::PATZ1‐fusion gene in pediatric high‐grade glioma (Laemmerer et al. 2025), with demonstrated cellular ALT features. Importantly, the telomeric replication mode within individual cancer biopsy is usually hard to address a clear‐cut mode of telomere replication, such that a heterogeneous appearance of telomeres is usually a strong ALT diagnostic indicator while how many of those cancer cells maintain their telomere integrity during BIR‐homologous recombination remain unresolved (Barthel et al. 2017). This portion of cancer cases especially warrant further detailed mechanistic studies to unveil whether at least some aspects of ALT like telomeric homologous recombination are utilized in those cells without apparent reactivation of telomerase. A recent human embryonic stem cell study further challenged the universality of the “two‐hits” hypothesis by demonstrating that disruption of the p53 and Rb pathways during cell differentiation in combination with ATRX loss‐of‐function alone is sufficient to induce all hallmarks of ALT without a “secondary” genetic hit (Turkalo et al. 2023). Therefore, during human cancer pathogenesis, continuous telomere instability combined with some aspects of DNA damage responses defects might be sufficient to render ATRX/DAXX mutant cells functionally immortalized with ALT‐like features.

### 3.2 Bridging Telomere Chromatin Status to DNA Replication Machinery

How the specific chromatin features of telomeres lead to the recruitment of break‐induced recombination enzymes and the formation of APBs is an active and intensive area of research. However, delineating a clear‐cut pathway remains challenging, primarily because capturing the full dynamic chromatin state of a telomere before it either forms a de novo APB or is recruited to an existing one is exceedingly difficult. Nonetheless, once a cell adopts the ALT pathway, its telomeres can be maintained over numerous cell cycles. This persistence suggests that ALT telomeres become functionally “locked” into this state through feedforward loops that ensure the sustained recruitment of all necessary components of the ALT replication machinery. This section will explore the known and potential connections between ALT‐specific telomere chromatin features and the subsequent cascade of events—the “ALT avalanche”—that they initiate.

#### 3.2.1 TRIM24‐Dependent ALT Cascade Initiation: From Replication Stress Sensing to ALT Platform Assembly

A hallmark of ALT telomeres, distinct from those without ALT features, is their hypersensitivity to replication stress. This can be triggered by various agents that stabilize G‐quadruplex structures or stall replication forks. Under such stress, DNA double‐strand breaks and the corresponding repair responses are readily induced at ALT telomeres. Similarly, recruiting a DNA endonuclease to telomeres through modules such as the TRF1‐FokI fusion protein also induces strong DNA damage responses (Tang et al. 2013).

**FIGURE 3:**
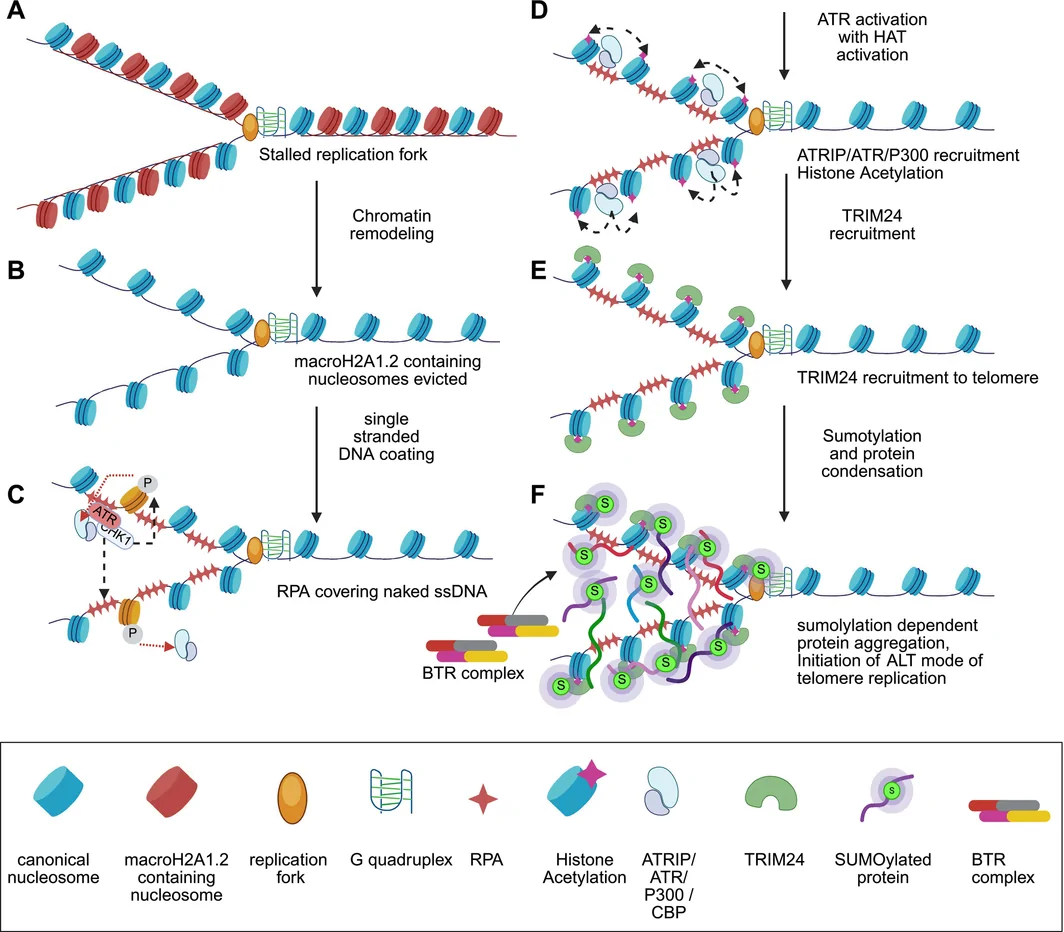
TRIM24‐mediated initiation of ALT. (A) The initiation of ALT is triggered by replication stress arising from stalled replication forks at telomeres such as G quadruplex containing DNA structures. (B) Replication stress induces chromatin remodeling, leading to the eviction of nucleosomes containing macroH2A1.2 and the exposure of single‐stranded DNA (ssDNA). (C) The exposed ssDNA can be quickly coated by RPA. RPA‐coated ssDNA recruits the ATR–ATRIP complex and activates ATR signaling. ATR signaling complex might bring along histone acetyltransferase P300/CBP; or P300/CBP enriched on the ATR activated telomeres via phosphorylated RPA mediated LLPS; or through ATR/chk1 mediated H3.3 S31 phosphorylation, which in turn strongly stimulate P300/CBP activity. (D) The histone acetyltransferase P300/CBP is responsible for depositing histone acetylation marks at ALT telomeres. (E) TRIM24 is specifically recruited to telomeric regions via its PHD (plant homeodomain) and BRD (bromodomain) domains, which recognize acetylated histone marks. (F) TRIM24 facilitates SUMOylation and protein condensation processes, forming SUMOylation‐dependent protein aggregates, including BTR complex responsible for the initiation of telomere recombination based DNA synthesis.

Recent studies pinpoint a specific association of the TRIM24 (tripartite motif‐containing 24) protein with ALT telomeres, which is absent from telomeres in non‐ALT cells, and its telomeric localization is exacerbated upon induction of telomeric replication stress (Kim et al. 2025) (Figure 3). As TRIM24 is a replication‐stress responder whose recruitment onto chromatin depends on ATR signaling and histone acetylation—particularly acetylation of H3K23, catalyzed by the histone acetyltransferase p300/CBP—these findings suggest that endogenous replication stress on ALT‐prone telomeres (potentially arising from G‐quadruplex‐rich telomeric sequences) is readily sensed and translated into TRIM24 recruitment. We also know that telomeric nucleosomes containing histone macroH2A1.2 are extremely sensitive to depletion upon replication stress, a process that is exacerbated in the absence of ATRX/DAXX (Kim et al. 2019) (Figure 3B). A speculative scenario therefore arises in which the eviction of nucleosomes leaves behind naked DNA, which in turn likely attracts large amounts of RPA (replication protein A) to bind the single‐stranded regions of exposed telomeres (Figure 3C). RPA binding to ssDNA has been linked to dynamic condensate functions (Spegg et al. 2023). Since ATR is primarily recruited to RPA‐coated single‐stranded DNA via ATRIP (Choi et al. 2010), nucleosome‐depleted telomeres provide promising substrates for potentiating ATR signaling (Figure 3D). Independent evidence indicates that in the context of ATRX inactivation, trapped proteins can promote ALT activity without a secondary genetic hit (Rose et al. 2023). These trapped proteins essentially act as potent replication blockers. Therefore, such blockade may further evict macroH2A1.2‐containing nucleosomes, driving local ATR signaling beyond a critical threshold.

While the replication stress at ALT telomere is known to drive TRIM24 recruitment via ATR signaling, detailed mechanistic linkage between ATR signaling and TRIM24 recruitment remains unclear. It is known, however, that the histone acetyltransferase p300/CBP and ATR can be found within the same signaling complex (Figure 3D), which also includes the ATR downstream kinase CHK1 (Stauffer et al. 2007). Whether ATR activation on naked DNA and acetylation of neighboring nucleosomes occur simultaneously via such signaling complex remains to be determined. Current experimental evidence indicates that TRIM24 recruitment is mediated through its reader domain recognizing H3K23ac deposited by p300/CBP (Kim et al. 2025) (Figure 3E). In addition, this recruitment occurs at sites of ATR‐dependent replication stress, suggesting a tight spatial coupling between these processes. Furthermore, RPA itself is a critical substrate for phosphorylation by ATR (Vassin et al. 2009), and DNA damage‐induced RPA hyperphosphorylation has been implicated in modulating cellular DNA dynamics (Patrick et al. 2005), suggesting that RPA phosphorylation likely plays a key role in resolving replication stress following ATR activation. In vitro biochemical evidence shows that phosphorylation of RPA, particularly within its N‐terminal intrinsically disordered region (IDR), influences RPA‐mediated protein condensation and its dynamic exchange once bound to single‐stranded DNA substrates (Spegg et al. 2023). Thus, it remains a plausible possibility that p300/CBP recruitment could be achieved through multicomponent protein phase separation involving RPA at stress foci, after the ATR/CHK1 cascade has fully executed its functions. An independent potential point of linking ATR/CHK1 signaling cascade to histone acetylation might be through histone modification itself. It is known that CHK1‐driven histone H3.3 serine 31 phosphorylation (serine 31 is unique to histone variant H3.3) (Hake et al. 2005) occurs, whose occurrence is important for survival of human ALT cancer cells (Chang et al. 2015). In the case of ATRX/DAXX deficient ALT cells, HIRA mediated H3.3 containing nucleosome loading is tightly coupled with CHK1 dependent H3.3 S31 phosphorylation (Lynskey et al. 2024). H3.3S31ph in a way robustly transforms the H3.3‐containing nucleosome from a stable state to a more dynamic configuration (Ma et al. 2025), highly prone to spontaneous nucleosome depletion. Furthermore, H3.3 S31Ph was discovered to be directly involved in stimulating histone acetyltransferase CBP/P300 activities on chromatin harboring active transcription (Martire et al. 2019). Therefore, it is likely that HIRA/ATR/CHK1 signaling hub on ALT telomeres can facilitate p300/CBP‐mediated histone acetylation via first depositing H3.3 S31Ph chromatin marks. Experimental approaches blocking either RPA or H3.3 S31 phosphorylation will help to identify whether there is a critical intermediate linking ATR activation on ALT telomeres to local CBP/P300 activity boost.

**FIGURE 4:**
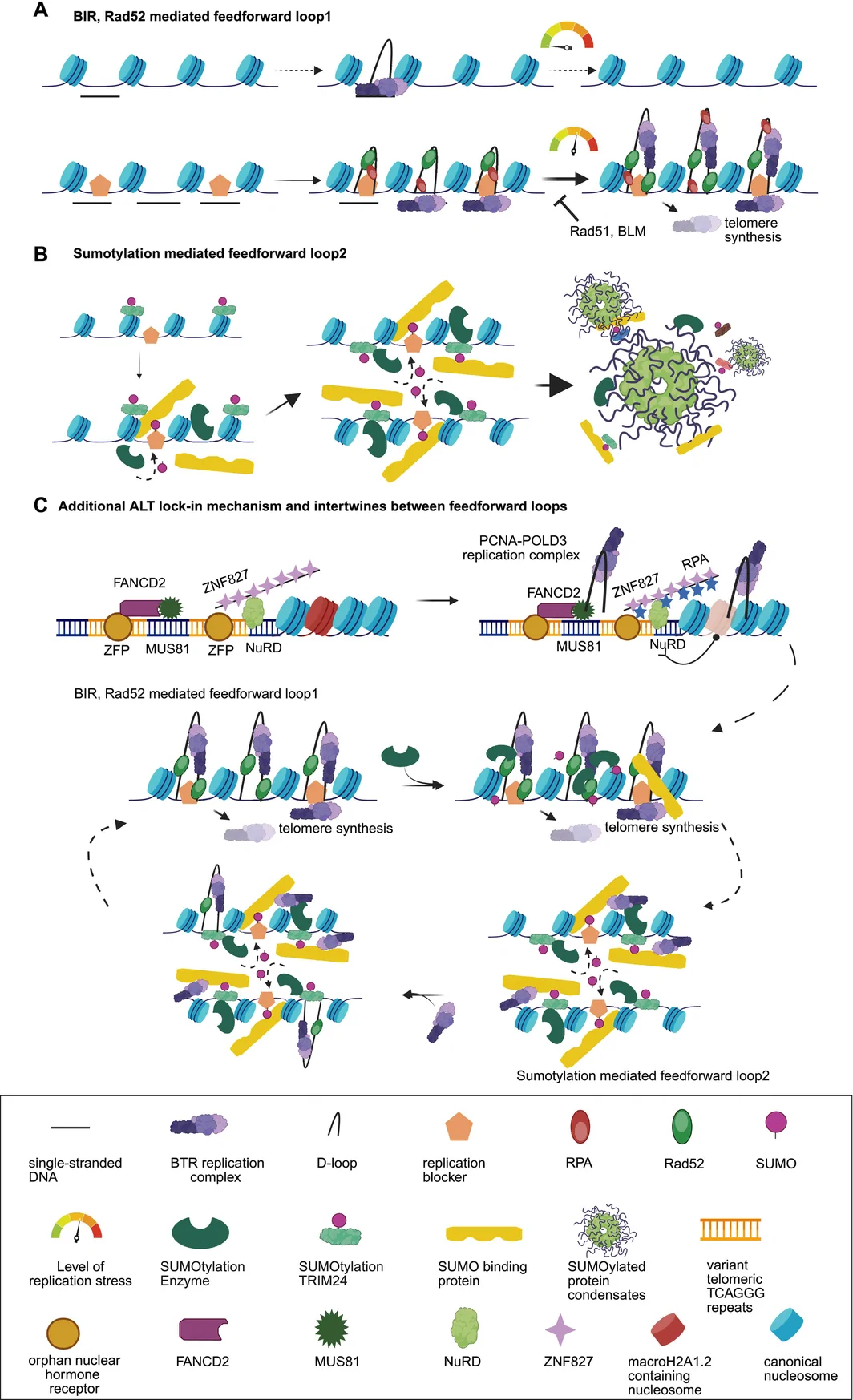
Three intertwined feedforward loops lock in the ALT state. (A) BIR and Rad52 Mediated Feedforward Loop 1: In response to replication stress on ALT telomeres, the BTR complex is recruited. Below a critical threshold of replication stress, BTR complex facilitates telomere synthesis without further exacerbating. When stress exceeds this threshold, the accumulated RPA‐coated ssDNA may form condensates that recruit more RAD52. RAD52 promotes excessive D‐loop formation, establishing a self‐amplifying feedforward cycle during telomere synthesis, which is modulated by braking factors including BLM helicase and RAD51. (B) SUMOylation mediated Feedforward Loop 2: In response to telomeric replication stress, TRIM24 recruits SUMOylation Enzyme, catalyzing widespread local protein SUMOylation. The SUMOylated proteins, together with proteins with SUMO‐binding motifs, assemble into stable condensates via multivalent weak protein–protein interactions. These condensates create a self‐sustaining microenvironment, or coagulate with preexisting condensates, thereby establishing feedforward loop 2 that locks in the ALT state. (C) Additional ALT lock‐in mechanism and interconnections between feedforward loops: Orphan nuclear hormone receptors bind to variant telomeric TCAGGG repeats. They recruit the zinc‐finger protein ZNF827 and the nucleosome remodeling and deacetylase (NuRD) complex. ZNF827/NuRD may also facilitate the eviction of macroH2A1.2‐containing nucleosomes. In parallel, these receptors interact with FANCD2, which recruits the MUS81 endonuclease for DNA break generation followed by PCNA–POLD3 replication complex to execute ALT synthesis. BTR complex mobilized onto ALT telomeres during the process is likely integrated into the BIR and Rad52 Mediated Feedforward Loop 1. Rad52, in turn, by recruiting SUMOylation enzymes, integrate the feedforward loop 1 with SUMOylation mediated Feedforward Loop 2. The recruitment of BTR complex onto SUMOylation‐dependent protein LLPS condensates, in turn, links feedforward loop 2 back to feedforward loop 1, forming an intertwined ALT state lock in network.

Downstream of ATR activation and p300/CBP‐mediated histone acetylation, TRIM24 occupies a critical position in the ALT cascade. Telomeric recruitment of TRIM24 is both necessary and sufficient for inducing ALT phenotypes, even in the absence of exogenous telomeric replication stress (Kim et al. 2025). Upon recruitment, TRIM24 employs its coiled‐coil domain to mediate the recruitment of ALT‐associated SUMO E3 ligases to telomeres, thereby facilitating its own SUMOylation as well as SUMOylation of other telomeric proteins (Figure 3F). Independently, the same coiled‐coil domain enables TRIM24 to efficiently recruit the major nuclear PML isoform PML‐IV to telomeres. PML‐IV serves as a core scaffold in endogenous APB formation, as it contains multiple SUMOylation sites along with two SIMs (SUMO‐interaction motifs). The multivalent, weak non‐covalent interactions between SUMO and SIM drive the assembly of SUMOylated protein condensates that constitute APBs. Thus, by simultaneously recruiting SUMO E3 ligases and their principal scaffold substrates, TRIM24 drives a local burst of SUMOylation that can either nucleate APBs de novo (Kim et al. 2025), or recruit preexisting SUMOylated protein condensates (Zhao et al. 2024) (Figures 3F and 4B). Importantly, once TRIM24 is sufficiently enriched at telomeres, it can even bypass the requirement for the scaffold PML‐IV in APB formation. In a SUMOylation‐dependent manner, concentrated TRIM24 can directly recruit the BTR complex. The functional independence of ALT from PML‐IV in this bypass strongly suggests that PML‐IV's role in endogenous APB formation is largely structural—orchestrating protein condensate assembly rather than executing core recombination functions. Indeed, when TRIM24, SUMO E3 ligases, and the BTR complex are simultaneously recruited at a telomere, efficient SUMOylated condensates can still form without PML‐IV involvement. While PML‐IV‐free APBs generated in this manner fully support ALT‐mediated telomere replication (Kim et al. 2025), TRIM24‐mediated APBs formed in the absence of BLM cannot sustain ALT, pinpointing the essential role of BLM within the BTR complex (which also includes POLD3, PCNA, and RAD52).

#### 3.2.2 A Self‐Sustaining BIR‐Mediated Feedforward Loop

The hierarchical ALT signaling cascade outlined above—from replication stress sensing to ATR signaling/histone acetylation and finally TRIM24‐dependent formation of SUMOylated protein condensates—remains incompletely understood and represents an oversimplified model of how telomeres adopt an ALT‐devoted replication state. Once established, ALT cells stably maintain this way of telomere replication, implying the existence of feedforward mechanisms that sustain the stable association of relevant BIR repair machinery at ALT telomeres.

Zhang et al. (Zhang et al. 2021) proposed a BIR‐mediated feedforward mechanism that stabilizes the association of the BTR break repair machinery at ALT telomeres. The trigger for ALT, endogenous replication stress, may originate from multiple sources, such as intrinsic telomeric G‐quadruplex structures or stable R‐loop replication intermediates (Figure 4A). The FANCM DNA translocase cooperates with the BLM–TOP3A–RMI (BTR) complex to resolve telomeric R‐loops (Lu et al. 2019; Silva et al. 2019; Pan et al. 2017). Therefore, it was originally thought that telomere association with BTR complexes effectively reduces local replication stress. However, it has been shown that APB‐associated functional BTR complexes instead enhance local ATR signaling, including increased RPA phosphorylation and the generation of additional telomeric DNA repair intermediates. Paradoxically, BLM, FANCM DNA translocase, and the POLD3 and POLD4 are involved in this APB‐dependent ATR signaling activation. This is achieved through efficient recruitment of RAD52 via BTR complex activity. Accumulation of RAD52 promotes further D‐loop formation on telomeric DNA (Zhang et al. 2019) (Figure 4A). Thus, once DNA replication stress surpasses a critical threshold and triggers ALT, it becomes self‐perpetuating within APBs. In addition to the well‐documented central role of Rad52 in D‐loop triggered BIR activity, a RAD52‐independent ALT pathway can proceed through first forming telomeric R‐loops by TERRA and RAD51AP1 (RAD51‐associated protein 1), and a subsequent R‐loop to D‐loop switch at telomeres to stimulate BIR (Yadav et al. 2022). Beyond interacting with TERRA, RAD51AP1 can participate in the BIR process by stabilizing RAD51‐ssDNA filaments and promoting DNA sister‐strand exchange (Kuhlen et al. 2025).

To prevent excessive replication stress accumulation at telomeres—this feedforward loop must be restrained by regulatory brakes. Without exhaustive prior investigations on such braking mechanisms, we will speculate a few hypothetical means ALT cells may employ to counterbalance the propagation of this feedforward cycle in the following section. Firstly, potential checkpoints may involve a critical local threshold of BIR activity under replication stress, which must be reached to enable efficient RAD52 recruitment to chromatin. This could be potentially mediated through the ability of RAD52 to incorporate into RPA protein condensates (Spegg et al. 2023), a process that itself requires a locally enriched concentration of RPA to initiate condensate formation. Therefore, when replication blocks are efficiently resolved via sub‐threshold BIR activity, no significant amount of RAD52 is recruited—thereby avoiding the exacerbation of preexisting replication stress (Figure 4A). Secondly, the extent of TERRA‐mediated R‐loops in telomeres is strongly associated with single‐stranded DNA synthesis, therefore need to be tightly regulated. In ALT cells when HIRA is depleted or ATR/CHK1 kinase is inhibited, H3.3S31ph is strongly diminished at telomeres, creating a highly accessible telomeric chromatin with excessive TERRA telomere transcription/enrichment, followed by massive ssTelo accumulations, resulting in nonproductive ALT and subsequent cell death (Lynskey et al. 2024). Therefore in ALT cells, maintaining a repressive, heterochromatin‐rich chromatin environment would be a third key for preventing excessive propagation of this feedforward loop, since unresolved TERRA R‐loops can in turn block HIRA‐mediated H3.3 deposition therefore pushing BIR‐centered replicative stress accumulation into a pit with no return (Lynskey et al. 2024). An additional heterochromatin residual safeguard mechanism to resolve excess replication stress utilizes so‐called CHAMP1 (chromosome alignment maintaining phosphoprotein 1) complex, made of CHAMP1, POGZ, HP1α, and SETDB1. CHAMP1 complex can be transiently recruited to stalled replication forks upon replication stress, where it facilitates replication fork stabilization via mediating H3K9me3 deposition, shielding the stalled fork from MRE11‐mediated DNA resecting (Li et al. 2026). Finally, ALT‐positive tumor cells frequently rely on boosting DNA translocase FANCM and SMARCAL1 activities to retreat the stalled replication fork, limiting replication stress buildup via BTR‐mediated feedforward loop (Lu et al. 2019; Halder et al. 2022; Fielden et al. 2025).

#### 3.2.3 SUMOylated Protein Mediated Liquid–Liquid Phase Separation and Coordinated Feedforward Loops Locking in the ALT State

Efficient telomeric DNA synthesis in ALT cells is strictly dependent on the presence of APBs (Zhang et al. 2019; Gaela et al. 2024). As previously discussed, APBs form primarily through LLPS (liquid–liquid phase separation), which achieves a locally high concentration of BIR repair machinery, including the BLM helicase. LLPS is a thermodynamically favorable process in cells; thus, preformed APBs tend to maintain/grow under steady‐state conditions by recruiting additional SUMOylated protein components or by fusing with preexisting PML bodies containing SUMOylated BIR components (Zhao et al. 2024). Consequently, once an APB reaches a critical size, the phase separation process becomes largely irreversible, establishing a second feedforward loop that drives ALT‐mediated telomere synthesis (Figure 4B). In non‐ALT cells, however, artificially tethering PML‐like SUMOylated protein condensates to telomeres does not trigger the ALT pathway (Min et al. 2019), reinforcing the view that PML functions largely as a structural scaffold within APBs without direct involvement in the ALT mechanism. Notably, when BLM with intact helicase activity is co‐overexpressed during the formation of such artificial condensates, robust ALT telomere synthesis is eminent (Min et al. 2019). This indicates that BLM acts as the critical effector that organizes the local BIR repair machinery to kickstart the first feedforward loop for ALT. Indeed, without a locally high concentration of BIR components—achieved within the protein condensate on telomeres—ALT fails to initiate. Since overcoming the inhibitory brakes to exacerbate local replication stress (restricting feedforward loop 1 as discussed in the previous section) may require continuously surpassing an activation threshold, LLPS can provide a precise, robust safety mechanism that locks the critical triggering agents at a steady, over‐threshold level. The above proposed interlocked relationship between phase separation and the propagation of replication stress is most clearly illustrated by manipulating SUMOylation‐dependent protein condensation. For example, As_2_O_3_ (arsenic trioxide) promotes PML degradation (Hands et al. 2014; Lallemand‐Breitenbach et al. 2012). Consequently, ALT‐positive cancer cells show greater sensitivity to arsenic trioxide‐induced cytotoxicity, a phenomenon associated with the disassembly of SUMOylated protein condensates in the nucleus (Gaela et al. 2024). Conversely, recruiting more of the SUMOylation scaffold protein PML to ALT telomeres significantly increases telomeric DNA replication stress, indicative of effective fueling feedforward loop 1 via LLPS (Kim et al. 2025). The intertwined relationships between the dual feedforward mechanisms can also be demonstrated by the contribution of BTR complex components in coalescing telomeres via phase separation, since the recruitment of RAD52 and the assembly of BIR machinery (driven by feedforward loop 1) enrich local SUMO‐dependent protein aggregates in turn (Zhao et al. 2024). This is potentially achieved by their stimulation of the SUMO ligases PIAS4 and MMS21 (Zhang et al. 2021), thereby supplying essential substrates for SUMOylation‐dependent LLPS (positive feedforward loop 2). Such reciprocal mechanistic link is also functionally critical, as the depletion of PIAS4 can significantly abolish the local accumulation of replication stress induced by RAD52/BIR. This suggests that feedforward loop 1 cannot perpetuate on its own without the “fuel” provided by feedforward loop 2 as well (Zhang et al. 2021) (Figure 4C).

Although the well‐studied protein SUMOylation mediated liquid–liquid phase separation can act as a potent organization hub propagating BIR related feedforward loop 1, there are potentially additional ALT telomere specific phase separation mechanisms connecting with increased local DNA replication stress. For example, ALT cells usually process higher TERRA transcript levels, which facilitate nucleic acid‐centered phase separation driven by protein‐RNA interactions between LSD1 and TERRA. G‐quadruplex structure dependent TERRA/LSD1 condensates formed on telomeric DNA also recruit the R‐loop stimulating protein Rad51AP1 and subsequently increase TERRA‐containing R‐loops at telomeres (Bhargava et al. 2022; Xu et al. 2024). While RAD51AP1 can trigger RAD52 independent R‐loop to D‐loop switch to stimulate BIR (Yadav et al. 2022). Both of those processes potentially link TERRA mediated phase separation back to fuel feedforward loop 1. The above‐mentioned nucleic acid‐centered phase separation process can be an independent but critical LLPS mechanism beyond those dependent on SUMOylation since depleting LSD1 in ALT cells alone can also lead to a reduction in APB numbers as well as a loss of ALT features (Xu et al. 2024). In addition, the heterochromatic features of ALT telomeres might also act as substrates for multivalent weak protein–protein interactions leading to telomere fusion since molecular tethering of HP1α at telomeres is sufficient to form fused telomere‐nuclear bodies in non‐ALT cells and even induce certain features of ALT‐like recombination (Taylor et al. 2026). Such observations indicate there might be multiple means of forming telomeric seeded LLPS structures and together promoting subsequent BIR‐mediated telomeric recombinations on the fused telomeric megastructure.

Beyond the two core, intertwined feedforward loops discussed above, additional “lock‐in” mechanisms likely operate once the ALT mode of telomere replication is established (Figure 4C). For instance, several orphan nuclear hormone receptor superfamily members—including COUP‐TF1, COUP‐TF2, TR2, TR4, and EAR2—specifically bind to variant telomeric TCAGGG repeats. These repeats are abundantly generated through ALT but are less abundant from normal telomeres (Conomos et al. 2012; Lee et al. 2014). Along with these receptors, FANCD2, a downstream effector of the Fanconi anemia pathway that is functionally linked to the FANCM complex (Walden and Deans 2014; O'Rourke et al. 2019; Xu et al. 2019), actively recruits the endonuclease MUS81 and promotes the loading of the PCNA–POLD3 replication complex onto ALT telomeres (Xu et al. 2019). In parallel, these nuclear receptors recruit the zinc‐finger protein ZNF827, along with the NuRD (nucleosome remodeling and histone deacetylation) complex (Gaela et al. 2024; Conomos et al. 2014). NuRD facilitates enhanced telomere–telomere interactions and promotes the loading of homologous recombination proteins necessary for ALT‐mediated DNA synthesis. Its histone deacetylation activity may also counterbalance CBP/p300‐dependent TRIM24 recruitment to ALT telomeres.

Notably, in non‐ALT cells lacking variant TCAGGG repeats, artificially tethering orphan nuclear hormone receptors to telomeres can induce substantial ALT‐like features, including telomere clustering, APB formation, and *de novo* C‐circle synthesis (Gaela et al. 2024). These effects likely involve chromatin remodeling processes and appear to act in concert with inactivation of the ATRX–DAXX chaperone complex.

Although how nuclear receptors shape the ALT telomeric chromatin remains to be fully appreciated, a plausible scenario is that ZNF827/NuRD participates directly in evicting nucleosomes containing histone macroH2A1.2 upon replication stress—a process known to be further facilitated by the absence of the ATRX–DAXX complex (Kim et al. 2019). Additionally, ZNF827 itself is a single‐stranded DNA‐binding protein that associates with RPA at stalled replication forks, which is the favorable substrate for activating the ATR–CHK1 DNA damage response pathway (Yang et al. 2024). Therefore, a subsequent coupling with NuRD‐dependent chromatin remodeling machinery via ZNF827 helps to reshape the local chromatin structures (Figure 4C). In parallel, orphan nuclear receptors facilitate heterochromatin maintenance on ALT telomeres by recruiting cofactors TRIM28 (tripartite motif‐containing 28), SETDB1, HP1, HDAC (histone deacetylase), and DNMT1 (DNA methyltransferase 1) to maintain repressive chromatin features (Tsai et al. 2026).

#### 3.2.4 How Does the Telomeric Chromatin Status Influence the Two Intertwined Feed Forward Mechanisms Uniquely Favored on ALT Telomeres?

The maintenance of both aforementioned feedforward loops is critically important for sustaining ALT‐mediated telomere replication, as supported by multiple lines of evidence. For instance, in ALT cancer cells, either depletion of PML or pharmacological inhibition of SUMOylation (Zhao et al. 2024) strongly suppresses telomere replication and cell viability. This demonstrates that phase‐separation‐dependent protein condensate formation (constituting positive feedforward loop 2) is essential for the ALT pathway. On the other hand, RAD52, as an essential signal amplifier for positive feedforward loop 1, is required for telomere maintenance in ALT‐positive cells (Zhang et al. 2019). A RAD52‐independent BIR pathway is activated only after telomere length is gradually reduced due to the attenuation of the RAD52‐dependent feedforward loop1.

It is important to note that while ALT telomeres must sustain a “chronic” state of replication stress and the associated DNA damage response (DDR) (Cesare et al. 2009), appropriate regulatory mechanisms must operate to prevent excessive DDR caused by the exponential accumulation of replication stress, which would otherwise lead to cell cycle arrest and cell death. For example, RAD51 is a key DDR factor that plays a central role in maintaining ALT telomere integrity, yet it is dispensable for break‐induced telomere synthesis (Dilley et al. 2016). Its primary function at ALT telomeres may be protecting stalled replication forks and suppressing the RAD52‐mediated feedforward loop (Zhang and Zou 2020) (Figure 4A). ALT telomeres may also employ protective strategies such as transcribing noncoding RNAs from sites of DNA damage (Pessina et al. 2019; Michelini et al. 2017). These transcripts promote the retention of DDR factors RAD51 and 53BP1 at telomeres (Rosso et al. 2023), since ablation of C‐rich telomeric RNAs in ALT cells triggers exacerbated RAD52‐dependent replication stress (Rosso et al. 2023). Similarly, RAD51 is required for telomere extension in cells overexpressing BLM, a key mediator of both feedforward loops (Sobinoff et al. 2017).

For ALT cancer cells, besides strategies to disarm those key braking mechanisms holding the telomeric replication stress under control, efficient therapeutics can best target either of the two interlocked feedforward loops. In the following section, we discuss whether the progression of these two interlocked feedforward loops can be modulated by the chromatin state of telomeres. If so, an ALT telomere–specific chromatin‐targeting therapy could selectively block ALT cancer cell proliferation without affecting other cells in vivo.

In ALT cells, the activation of KDM4B has been shown to inhibit APB formation and suppress the ALT pathway, with no corresponding effect observed in non‐ALT cells. Reactivation of KDM4B in these cells leads to its prominent recruitment to telomeres, subsequent loss of APBs, and elevated levels of DNA damage (Udugama et al. 2021). This strongly suggests that the ALT feedforward loop requires a heterochromatin‐rich genomic context, likely because sustained ATR activation depends on dynamic nucleosome turnover—a process potentially particularly feasible at heterochromatin‐rich telomeres (Kim et al. 2019). In cancer patients, KDM4B inactivation is mainly caused by histone mutation‐mediated inhibition or small molecule metabolite‐mediated inhibition (Udugama et al. 2021). Relieving such inhibitions can be achieved through therapeutic targeting of mutant IDH to reduce 2‐hydroxyglutarate level (Ye et al. 2018; Yen et al. 2017), or through using KDM4B small molecule agonists.

The establishment of both feedforward loops involves TRIM24 localization on telomeres, a process dependent on its PHD and BRD reader motifs binding to acetylated histones on ALT telomeres (Kim et al. 2025). Indeed, depleting CBP/p300 or CBP/p300 inhibitors leads to significant ALT telomere loss, driven by reduced telomere DNA synthesis and increased telomere fragility. Therefore, ALT feedforward loops depend on ALT‐specific histone acetylation, which is further amplified by subsequent telomeric DSBs and SUMOylation‐dependent protein condensation (Kim et al. 2025). Since CBP/p300 are the major cellular histone acetyltransferases responsible for various epigenetic processes such as transcriptional activation (Yazıcı and McIntyre 2025), systemic inhibition of CBP/p300 using small‐molecule inhibitors such as A‐485 may cause broad cytotoxic effects on normal tissues (Lasko et al. 2017). However, given the critical dependence of ALT on specific telomeric chromatin states (Gao and Pickett 2022), a more precise strategy, such as targeted deacetylation via site‐specific epigenetic editing, could be highly effective in disrupting ALT maintenance.

## 4 Summary and Outlook

Major advances have been made in understanding the complex physicochemical nature of human telomeric chromatin since the discovery of heterochromatin nearly a century ago. Further deciphering the mechanisms of chromatin homeostasis and dynamics at telomeres will help maintain telomere structure in healthy, long‐lived stem cells and extend the human health span. Conversely, uncovering how telomeric chromatin is rewired in disease states—such as in ALT‐positive cancer cells—offers an exciting and promising opportunity to selectively block cancer cell proliferation by disrupting its alternative chromatin architecture.

## Author Contributions

**Jie Wang:** conceptualization, writing – original draft, writing – review and editing, literature curation. **Yuehui Su:** visualization, writing – review and editing, literature curation. **Chao Chen:** writing – review and editing, literature curation, visualization. **Yueying Li:** writing – review and editing, visualization, validation. **Jiajia Han:** writing – review and editing, visualization, literature curation. **Liping Du:** writing – review and editing, validation. **Liyun Zheng:** writing – review and editing, validation. **Ya‐Long Dang:** conceptualization, writing – original draft, writing – review and editing. **Ying Peng:** conceptualization, writing – original draft, writing – review and editing, funding acquisition. All authors reviewed and approved the final version of the manuscript.

## Funding

This work was funded by Henan provincial key research and development grant (261111312100), the Key Scientific and Technological Project of Henan Province (262102310328), Henan Provincial Natural Science Foundation (262300420609), and Henan Institute of Pharmaceutic and Medical sciences Innovation team grant (2026BP0102).

## Conflicts of Interest

The authors declare no conflicts of interest.

## Supporting information


**Table S1:** List of abbreviations.

## Data Availability

The authors have nothing to report.
