# Supplementary material for: Twisting the End Game: How Telomere Chromatin Modifications Shape Telomere Maintenance
Source: Aging Cell. 2026 Sep 6;25(9):e70694. doi: 10.1111/acel.70694 (PMC13546678; doi:10.1111/acel.70694)
Supplement: Supplementary file 1 [file ACEL-25-e70694-s001.pdf]

Supplementary Table 1. List of abbreviations		
**Number**	**Abbreviation**	**Full Term**
1	ALT	alternative lengthening of telomeres
2	APB	ALT-associated promyelocytic leukemia bodies
3	ASF1a/b	anti-silencing function 1A/1B histone chaperones
4	ATM	ataxia telangiectasia mutated
5	ATR	ataxia telangiectasia and Rad3-related kinase
6	ATRIP	ATR-interacting protein
7	ATRX	α-thalassemia X-linked mental retardation protein
8	BIR	break-induced replication
9	BLM	Bloom syndrome helicase
10	BRD	bromodomain
11	BTR	BLM-TOP3A-RMI dissolvase complex
12	CBP	CREB binding protein
13	CHAMP1	chromosome alignment maintaining phosphoprotein 1
14	CHK1	checkpoint kinase 1
15	COUP-TF1/2	chicken ovalbumin upstream promoter-transcription factor 1/2
16	DAXX	death domain associated protein
17	D-loop	displacement loop
18	DNMT1	DNA methyltransferase 1
19	DTD	double Tudor domain
20	EAR2	V-erbA-related protein 2
21	ERCC4	excision repair cross-complementing group 4
22	FANCD2	Fanconi anemia complementation group D2
23	FANCM	Fanconi anemia complementation group M
24	G9a	euchromatic histone-lysine N-methyltransferase 2
25	GBM	glioblastoma multiforme
26	GLP	G9a-like protein
27	H3.3 S31P	phosphorylation of serine 31 on histone variant H3.3
28	HDAC	histone deacetylase
29	HIRA	histone cell cycle regulation defective homolog A
30	HMT	histone methyltransferase
31	HP1	heterochromatin protein 1
32	IDH1	isocitrate dehydrogenase 1
33	KDM	histone lysine demethylase
34	LLPS	liquid-liquid phase separation
35	LSD1	lysine-specific demethylase 1A

36	MLL1	mixed lineage leukemia 1
37	MMS21	methyl methanesulfonate sensitivity protein 21
38	MUS81	MUS81 structure-specific endonuclease
39	NuRD	nucleosome remodeling and histone deacetylation complex
40	PARG	poly(ADP-ribose) glycohydrolase
41	PARP	poly(ADP-ribose) polymerase
42	PARPi	PARP inhibitor
43	PCNA	proliferating cell nuclear antigen
44	PEV	position effect variegation
45	PHD	plant homeodomain
46	PIAS4	protein inhibitor of activated STAT 4
47	PML	promyelocytic leukemia protein
48	PML-IV	PML isoform IV
49	POGZ	pogo transposable element derived protein with zinc finger domain
50	POLD3/4	DNA polymerase delta subunit 3/4
51	POT1	protection of telomeres 1
52	RAD51	RAD51 recombinase (radiation sensitive 51)
53	RAD51AP1	RAD51-associated protein 1
54	RAD52	RAD52 DNA repair protein (radiation sensitive 52)
55	RAP1	repressor/activator protein 1
56	R-loop	RNA-DNA hybrid loop
57	RMI	RecQ-mediated genome instability protein
58	RPA	replication protein A
59	SETDB1/2	SET domain bifurcated histone lysine methyltransferase 1/2
60	SIM	SUMO-interaction motif
61	SLX1	SLX1 structure-specific endonuclease subunit
62	SLX4	SLX4 structure-specific endonuclease scaffold protein
63	SMARCAL1	SWI/SNF-related matrix-associated actin-dependent regulator of chromatin subfamily A-like protein 1
64	Su(var)3-9	suppressor of variegation, on chromosome 3, #9
65	SUMO	small ubiquitin-like modifier
66	SUV39H1/2	suppressor of variegation 3-9 homolog 1/2
67	TERRA	telomeric repeat-containing RNA
68	TIN2	TRF1-interacting nuclear protein 2
69	TOP3A	topoisomerase III alpha
70	TPE	telomere position effect
71	TPP1	TIN2-interacting protein (gene symbol ACD)
72	TR2	testicular receptor 2

73	TR4	testicular receptor 4
74	TRF1/2	telomere repeat binding factor 1/2
75	TRIM24	tripartite motif-containing 24
76	TRIM28	tripartite motif-containing 28
77	ZNF827	zinc finger protein 827
